# Übergewicht und Adipositas bei Erwachsenen: allgemeine Behandlungsgrundsätze und konservatives Management

**DOI:** 10.1007/s00508-023-02270-9

**Published:** 2023-10-11

**Authors:** Johanna Maria Brix, Barbara Andersen, Kadriye Aydinkoc-Tuzcu, Philipp Beckerhinn, Agnes Brossard-Eitzinger, Anna Cavini, Christian Ciardi, Martin Clodi, Marlies Eichner, Brigitte Erlacher, Markus Fahrnberger, Daniel Moritz Felsenreich, Claudia Francesconi, Bettina Göbel, Elisabeth Hölbing, Friedrich Hoppichler, Joakim Huber, Simone Leonora Huber, Bianca Karla Itariu, Birgit Jandrasitz, Florian W. Kiefer, Gerd Köhler, Renate Kruschitz, Bernhard Ludvik, Andrea Malzner, Alexander Moosbrugger, Anna Öfferlbauer-Ernst, Verena Parzer, Gerhard Prager, Michael Resl, Claudia Ress, Christian Schelkshorn, Thomas Scherer, Harald Sourji, Lars Stechemesser, Thomas Stulnig, Hermann Toplak, Maria Wakolbinger, Alexander Vonbank, Daniel Weghuber

**Affiliations:** 1 1. Medizinische Abteilung mit Diabetologie, Endokrinologie und Nephrologie, Karl Landsteiner Institut für Adipositas und Stoffwechselerkrankungen, Klinik Landstraße, Wien, Österreich; 2 Psychologische Praxis psychologie-andersen.at, Wien, Österreich; 3 5. Medizinische Abteilung für Endokrinologie, Rheumatologie und Akutgeriatrie, Klinik Ottakring, Wien, Österreich; 4 Abteilung für Chirurgie, Landesklinikum Hollabrunn, Hollabrunn, Österreich; 5 Universitätsklinik für Innere Medizin I, mit Gastroenterologie Hepatologie, Nephrologie, Stoffwechsel und Diabetologie, Uniklinikum der Paracelsus Medizinischen Privatuniversität, Salzburg, Österreich; 6 kokon – Reha für junge Menschen, Bad Erlach, Österreich; 7 Abteilung für Innere Medizin, Krankenhaus St. Vinzenz Zams, Zams, Österreich; 8 ICMR – Institute for Cardiovascular and Metabolic Research, Johannes Kepler Universität Linz, Linz, Österreich; 9 3. Medizinische Abteilung mit Stoffwechselerkrankungen und Nephrologie, Karl Landsteiner-Institut für Stoffwechselerkrankungen und Nephrologie, Klinik Hietzing, Wien, Österreich; 10 Abteilung Innere Medizin III, Krankenhaus Barmherzige Schwestern, Wien, Österreich; 11 Sonderkrankenanstalt Rehabilitationszentrum Alland, Alland, Österreich; 12 Klinische Abteilung für Viszeralchirurgie, Universitätsklinik für Allgemeinchirurgie, Medizinische Universität Wien, Wien, Österreich; 13 Landeskrankenhaus Hochsteiermark, Standort Leoben, Leoben, Österreich; 14 Abteilung für Innere Medizin, Krankenhaus der Barmherzigen Brüder Salzburg, Salzburg, Österreich; 15 Interne Abteilung mit Akutgeriatrie und Palliativmedizin, Franziskus Spital, Standort Landstraße, Wien, Österreich; 16 Klinische Abteilung für Endokrinologie und Stoffwechsel, Universitätsklinik für Innere Medizin III, Medizinische Universität Wien, Wien, Österreich; 17 Rehabilitationszentrum Aflenz für Stoffwechselerkrankungen mit Schwerpunkt Diabetes mellitus und hochgradige Adipositas, Aflenz, Österreich; 18 Abteilung für Innere Medizin, Krankenhaus der Elisabethinen Klagenfurt, Klagenfurt, Österreich; 19 Abteilung für Innere Medizin I, Klinikum Wels Grieskirchen, Standort Wels, Wels, Österreich; 20 Abteilung für Innere Medizin II, Konventhospital der Barmherzigen Brüder Graz-Marschallgasse, Graz, Österreich; 21 Department für Innere Medizin I, Medizinische Universität Innsbruck, Innsbruck, Österreich; 22 1. Medizinische Abteilung, Landesklinikum Stockerau, Stockerau, Österreich; 23 Klinische Abteilung für Endokrinologie und Diabetologie, Medizinische Universität Graz, Graz, Österreich; 24 Abteilung für Sozial- und Präventivmedizin, Zentrum für Public Health, Medizinische Universität Wien, Wien, Österreich; 25 Abteilung für Innere Medizin I, Akademisches Lehrkrankenhaus Feldkirch, Feldkirch, Österreich; 26 Universitätsklinik für Kinder- und Jugendheilkunde, Uniklinikum der Paracelsus Medizinischen Privatuniversität, Salzburg, Österreich; 27 Abteilung für Innere Medizin mit Diabetologie, Gastroenterologie und Hepatologie, Rheumatologie und Intensivmedizin, Konventhospital der Barmherzigen Brüder Linz, Linz, Österreich; 28 Institut SIPCAN – Initiative für ein gesundes Leben, Salzburg, Österreich

**Keywords:** Adipositas, Diagnose, Konservative Therapie, Lebensstil, Medikamente, Obesity, Diagnosis, Conservative treatment, Lifestyle, Medication

## Abstract

Die Prävalenz von Übergewicht und Adipositas nimmt in Österreich – wie auch international – kontinuierlich zu. Insbesondere Adipositas ist mit multiplen Gesundheitsrisiken, Begleiterkrankungen, funktionellen Einschränkungen und sozialer Stigmatisierung assoziiert. Adipositas ist eine eigenständige und komplexe chronische Erkrankung und entsprechend multidisziplinär durch qualifizierte Fachkräfte zu behandeln. In Ergänzung rezenter internationaler Leitlinien skizziert das vorliegende Konsensuspapier allgemeine Grundsätze des Managements von Übergewicht und Adipositas und gibt Handlungsanleitungen für die Diagnose und für die konservative Therapie, wobei der Schwerpunkt auf die Lebensstilmodifikation und die medikamentöse Gewichtskontrolle gelegt wird. Anhand des „5A“-Modells der Verhaltensintervention wird ein Handlungsleitfaden für eine strukturierte, praxisorientierte und PatientInnen-zentrierte Betreuung von Menschen mit Übergewicht und Adipositas in Österreich präsentiert.

## Abstract (en)

The prevalence of overweight and obesity is steadily increasing in Austria as well as internationally. Obesity in particular is associated with multiple health risks, comorbidities, functional disability, and social stigma. Obesity is an independent, complex, chronic disease and should be treated as such by a multidisciplinary team of appropriately qualified personnel. In addition to recent international guidelines, this consensus paper outlines the overall principles of the management of overweight and obesity and provides guidance for the diagnosis and conservative treatment, focusing on lifestyle modifications and pharmacotherapy. Using the “5A” framework of behavioral health intervention, guidelines for a structured, pragmatic, and patient-centered medical care of adults with overweight or obesity are presented.

Adipositas ist eine durch übermäßiges Körperfett charakterisierte chronische Erkrankung, die mit einer Beeinträchtigung des Allgemeinzustands und der Lebensqualität, körperlichen und psychischen Komorbiditäten sowie einer reduzierten Lebenserwartung assoziiert ist [1–3]. Nach Angaben der Weltgesundheitsorganisation (WHO) waren im Jahr 2016 weltweit 1,9 Mrd. Menschen (39 % der Weltbevölkerung ab 18 Jahren) von Übergewicht, davon 650 Mio. Menschen (13 % der erwachsenen Weltbevölkerung) von Adipositas mit einem Body Mass Index (BMI) von ≥ 30 kg/m^2^ (Tab. 1) betroffen [4]. In Österreich hatte im Jahr 2019 jede dritte Person im Alter ab 15 Jahren einen BMI zwischen 25 und 30 kg/m^2^, die Adipositasprävalenz lag bei 16,5 % [5]. Seit 1980 hat sich die Zahl der Menschen mit Adipositas weltweit verdoppelt [4]. In den nächsten Jahren wird ein weiterer Anstieg der Adipositasprävalenz, in Österreich auf 23 % bis 2030, prognostiziert [6, 7].

**Table 1:** 

ICD-Code	Klassifizierung	Body Mass Index (BMI)
XS11	Untergewicht	< 18,5 kg/m^2^
XS43	Normalgewicht	18,5–24,9 kg/m^2^
XS7R	Prä-Adipositas	25,0–29,9 kg/m^2^
XS3Y	Adipositas, Klasse I	30,0–34,9 kg/m^2^
XS6N	Adipositas, Klasse II	35,0–39,9 kg/m^2^
XS2B	Adipositas, Klasse III	≥ 40 kg/m^2^

Nach Berechnungen der Global Burden of Disease 2015 Obesity Collaborators stehen weltweit rund 4 Mio. Todesfälle jährlich im Zusammenhang mit hohen BMI-Werten, davon 40 % bei Menschen mit Übergewicht [8]. Laut WHO sind weltweit 44 % der Diabetesfälle, 23 % der Fälle von ischämischer Herzkrankheit und 7–44 % der Krebserkrankungen auf Übergewicht bzw. Adipositas zurückzuführen. Damit sind Übergewicht und Adipositas auf globaler Ebene derzeit die fünfthäufigste Todesursache [4].

Die hohe Prävalenz von Begleit- und Folgeerkrankungen (Tab. 2) und die häufig beeinträchtigte Berufsfähigkeit und Alltagskompetenz der Betroffenen machen Übergewicht und Adipositas zur wachsenden volksgesundheitlichen und volkswirtschaftlichen Herausforderung. Menschen mit Adipositas sehen sich darüber hinaus oft mit Stigmatisierung und sozialer Ausgrenzung konfrontiert, was zur erhöhten Morbidität und Mortalität beiträgt [1–3].

**Table 2:** 

*Zu Übergewicht- bzw. Adipositas-assoziierten Erkrankungen zählen insbesondere:*
Diabetes mellitus Typ 2
Arterielle Hypertonie
Dyslipidämie
Kardiovaskuläre Erkrankungen
Nichtalkoholische Fettlebererkrankung
Gastroösophageale Refluxkrankheit
Polyzystisches Ovarialsyndrom
Belastungsharninkontinenz
Obstruktives Schlafapnoesyndrom
Arthrosen und andere degenerative Erkrankungen des Stütz- und Bewegungsapparats
Tumorerkrankungen
Essstörungen (z. B. Binge-Eating, Night-Eating, Problem-Eating, Snacking, Nibbling)
Psychische Erkrankungen (z. B. Depression, Angststörungen, bipolare Störungen, Autoaggression)

## Grundlagen von Übergewicht und Adipositas

Übergewicht und Adipositas entstehen in einem komplexen Zusammenspiel von (epi)genetischen, hormonellen, verhaltensbiologischen, psychologischen, kulturellen und sozioökonomischen Faktoren. Dazu kommen Gewichtszunahmen aufgrund von anderen Primärerkrankungen oder Medikamenten [9, 10]. Die Pathogenese ist meist polygenetisch; monogene Formen (z. B. auf Basis einer Melanocortin-4-Rezeptor‑, Leptin- oder Leptinrezeptor-Mutation) sind eher selten [11].

Unmittelbare biomedizinische Ursache der Gewichtszunahme ist ein anhaltendes Ungleichgewicht zwischen dem physiologischen Energiebedarf (Tab. 3) und der Energiezufuhr über die Nahrungsaufnahme. Weltweit hat der Konsum von energie-, fett- und zuckerreichen Nahrungsmitteln zugenommen, während körperliche Aktivität und Energieverbrauch zurückgegangen sind (z. B. aufgrund der sitzenden Tätigkeiten vieler Arbeitsformen, veränderter Transportmittel und zunehmender Urbanisierung). Modifikationen in den Ernährungs- und Bewegungsmustern sind oft das Ergebnis sich entwickelnder sozialer und umweltbedingter Veränderungen und des Mangels an unterstützenden Maßnahmen in Bereichen wie Gesundheit, Landwirtschaft, Verkehr, Urbanisierung bzw. Stadtplanung, Umwelt, Lebensmittelverarbeitung, Vertrieb, Marketing, Medien und Bildung [12].

**Table 3:** 

Der physiologische Energiebedarf (Tagesgesamtenergieumsatz, „total energy expenditure“ [TEE]) setzt sich zusammen aus: – Grundumsatz – Nahrungsinduzierte Thermogenese – Körperliche Aktivität
Der **Grundumsatz** (G) kann nach der Harris-Benedict-Formel berechnet werden. Das Ergebnis ist ein grober Richtwert für den täglichen Energiebedarf: – Frauen: *G [kcal]* *=* *655* *+* 9,6 * KG *+* 1,8 * L *−* 4,7 * A – Männer: *G [kcal]* *=* *66,5* *+* 13,7 * KG *+* 5,0 * L *−* 6,8 * A KG: Körpergewicht [kg]; L: Körpergröße [cm]; A: Alter [Jahre]
Der Tagesgesamtenergie**umsatz** (TEE) wird näherungsweise berechnet als – *TEE* *=* Grundumsatz * PAL Für den Aktivitätsfaktor („physical activity level“ [PAL]) wurden empirisch folgende Werte bestimmt: –Körperlich inaktiv: ≥ 1,0 bis < 1,4 – Körperlich wenig aktiv: ≥ 1,4 bis < 1,6 – Körperlich aktiv: ≥ 1,6 bis < 1,9 – Körperlich sehr aktiv: ≥ 1,9 bis < 2,5

Therapeutisch beeinflussbare Komponenten des Energiebedarfs sind v. a. das Ausmaß der körperlichen Aktivität sowie (indirekt) der Grundumsatz, der neben Geschlecht und Alter insbesondere von der Muskelmasse abhängt [13]. Die Energiezufuhr unterliegt zahlreichen endogenen und exogenen Regelkreisen: Die physiologische Regulation der Nahrungsaufnahme wird zentral im Hypothalamus und im Hirnstamm koordiniert. Periphere Signale liefern Information über die Menge und Beschaffenheit der aufgenommenen Nahrung und über den Füllstand der körpereigenen Energiedepots; sie wirken entweder stimulierend (orexigen; Ghrelin) oder hemmend (anorexigen; z. B. Leptin, „glucagon-like peptide 1“ [GLP-1], Amylin) auf das Hungerempfinden. Zusätzlich werden sensorische Reize (z. B. Geschmack, Geruch und Aussehen von Nahrung) aus anderen Hirnregionen verarbeitet. Die physiologische Regulation der Nahrungsaufnahme wird moduliert durch verhaltensbiologische und psychologische/psychiatrische Faktoren (z. B. Essen als Belohnung oder Kompensation negativer Empfindungen, Essstörungen, Suchtverhalten), soziale Konventionen und Zwänge und schließlich durch die exekutive Hirnfunktion, d. h. die bewusste Entscheidung für oder gegen das Essen [10, 14–16].

Menschen mit Normalgewicht können sich adaptiv an Schwankungen von Energiebedarf und Energiezufuhr anpassen und sind dadurch in der Lage, das Körpergewicht stabil zu halten. Umgekehrt ist die Dysregulation der Energiehomöostase essenziell für die Entstehung von Adipositas. Eine zentrale Rolle spielt dabei die viszerale Fettmasse, die bei Normalgewicht inhibierend, mit zunehmender Adipositas hingegen stimulierend auf Hunger und Appetit wirkt – ein Effekt, der durch eine sedentäre Lebensweise offenbar verstärkt wird. Inflammatorische Prozesse im viszeralen Fettgewebe und in den Hunger-Sättigung-Zentren des Gehirns tragen zusätzlich zur Entkopplung des Appetits vom physiologischen Energiebedarf bei, wobei es zum „Reset“ des endogen determinierten Zielgewichts zu kommen scheint, d. h. der Körper versucht, ein einmal erreichtes Höchstgewicht zu halten bzw. wieder zu erreichen. Dieser in der Literatur als „Set-point-Theorie“ [17] bekannte Prozess erklärt zumindest teilweise die Schwierigkeiten beim Versuch, dauerhaft abzunehmen, verdeutlicht aber auch die Notwendigkeit der chronischen Betreuung von Menschen mit Adipositas, um neuerlichen Gewichtszunahmen vorzubeugen [10, 15, 18].

## Klassifikation und Diagnose

Nach der aktuellen ICD-11-Klassifikation der WHO (2023) wird Adipositas (5B81) über den BMI definiert (Tab. 1; [19]). Fachgesellschaften wie die American Association of Clinical Endocrinologists (AACE, 2017) und die European Association for the Study of Obesity (EASO, 2019) kritisieren die BMI-zentrierte Kodierung als inadäquat und schlagen ein Vorgehen vor, das neben anthropometrischen Daten und der Pathogenese auch Adipositas-assoziierte Komplikationen und damit verbundene Risiken berücksichtigt. Dahinter steht die Überlegung, dass der BMI abhängig von ethnischer Zugehörigkeit, Alter, Körperbau und Gesundheitszustand stark variiert und z. B. bei SportlerInnen mit großer Muskelmasse oder bei älteren, sarkopenischen Menschen keine verlässliche Auskunft über den Körperfettgehalt gibt [20, 21].

**Figure 1:**
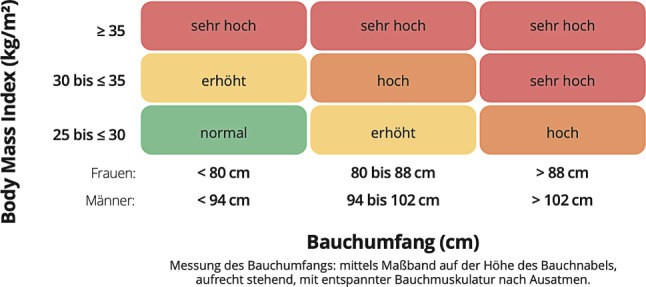

Eine viszerale Adipositas liegt bei einem BMI > 30 kg/m^2^ fast immer, bedingt durch eine ungünstige Relation von Fett- und Muskelgewebe, aber auch bei einem Drittel der gemäß BMI-Wert Personen mit Normalgewicht vor [22]. Trotzdem bleibt der BMI ein einfaches und breit verfügbares Screeningtool. Besser als der BMI korreliert der Taillenumfang („waist circumference“) mit der intraabdominalen Fettmenge. Der Taillenumfang soll standardisiert mit einem flexiblen, nicht elastischen Maßband in der Mitte zwischen der untersten Rippe und dem Beckenkamm gemessen werden [23]. Für EuropäerInnen gelten Werte > 94 cm bei Männern bzw. > 80 cm bei nichtschwangeren Frauen als Hinweis auf einen erhöhten viszeralen Fettanteil, der ein erhöhtes kardiovaskuläres Risiko im Rahmen des metabolischen Syndroms wahrscheinlich macht; ab 102 cm (Männer) bzw. 88 cm (Frauen) hat der Taillenumfang durch das Ausmaß der viszeralen Fettmenge selbst Krankheitswert (Abb. 1; [24, 25]). Weitere Informationen über die viszerale Fettakkumulation und weitere Parameter der Körperzusammensetzung liefern das Verhältnis zwischen Taillen- und Hüftumfang („waist-to-hip ratio“) und gerätediagnostische Verfahren wie Magnetresonanztomographie, „dual energy X‑ray absorptiometry“ (DEXA) oder Bioimpedanzanalyse (BIA) [26]. Zunehmend an Bedeutung gewinnt die „waist to height ratio“, welche auch ein leicht zu kalkulierendes, gutes Maß für die viszerale Adipositas ist [27].

**Figure 2:**
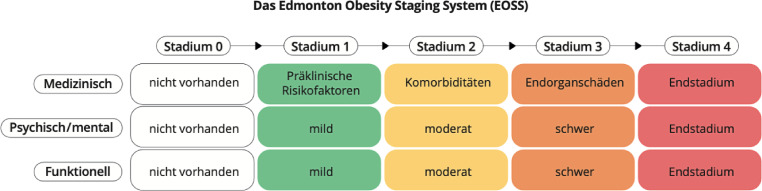

Auf Populationsebene ist der BMI als Prädiktor für die Morbidität und Mortalität von Menschen mit Übergewicht bzw. Adipositas gut etabliert [28]. Zur Abschätzung des individuellen gewichtsassoziierten kardiovaskulären Risikos kann eine Kombination aus BMI und Taillenumfang herangezogen werden (Abb. 1). Einen detaillierteren Blick auf die konkrete klinische Situation erlaubt das Edmonton Obesity Staging System (EOSS, Abb. 2; [29]). Mit dem EOSS kann der Schweregerad von Adipositas anhand von metabolischen, psychischen/mentalen und funktionellen Parametern objektiviert werden, und es können jene Personen identifiziert werden, die von einer therapeutischen Intervention besonders profitieren [30].

Neben Anthropometrie und Körperzusammensetzung inkludiert die klinische Untersuchung die Abklärung möglicher Begleiterkrankungen (Tab. 2) und das Erfassen spezifischer anamnestischer Informationen (Tab. 4, Empfehlungen 4.4).

**Table 4:** 

**Figure 3:**
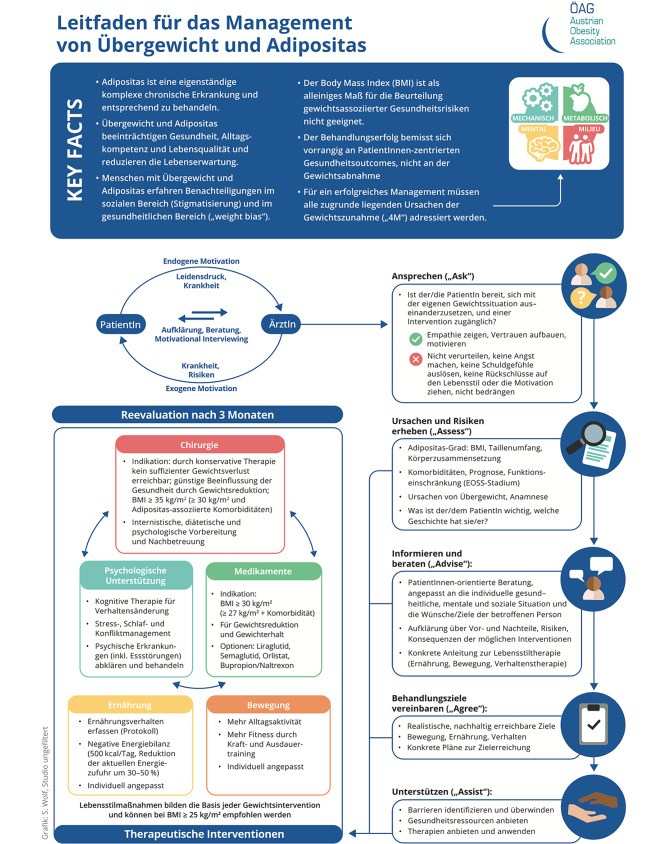

**4.1. Allgemeine Grundsätze**
Adipositas ist als eigenständige komplexe chronische Erkrankung einzuordnen und entsprechend zu behandeln
Die Betreuung von Menschen mit Übergewicht oder Adipositas soll multidisziplinär durch entsprechend qualifizierte Fachkräfte (ÄrztInnen, DiätologInnen, PhysiotherapeutInnen, SportwissenschaftlerInnen und ErnährungswissenschaftlerInnen mit entsprechender Zusatzausbildung sowie PsychotherapeutInnen bzw. klinische PsychologInnen oder GesundheitspsychologInnen) erfolgen
Der Umgang mit Menschen, die von Übergewicht oder Adipositas betroffen sind, soll respektvoll, empathisch und vorurteilsfrei erfolgen. Auf ihre Bedürfnisse und Ängste soll eingegangen werden
Die Art und Intensität der Gewichtsinterventionen sollen angepasst an das persönliche Risiko der Person mit Übergewicht bzw. Adipositas, an ihre Präferenzen, sozialen Umständen und Erfahrungen mit vorangegangenen Versuchen, Gewicht zu reduzieren, und an ihr Potenzial zur Verbesserung des Gesundheitszustands erfolgen
Die Betreuenden müssen sich des Aufwands, der notwendig ist, um Gewicht zu verlieren, Gewicht zu halten bzw. eine neuerliche Gewichtszunahme zu verhindern, und der Stigmatisierung, die Menschen mit Adipositas empfinden können, bewusst sein
Jede geplante Intervention soll mit den betroffenen Personen besprochen und ihr Einverständnis im Sinne eines „Shared decision-making“-Prozesses eingeholt werden
Das soziale Umfeld der betroffenen Personen soll, soweit möglich und gewünscht, unterstützend in die Betreuung miteinbezogen werden
**4.2. Diagnose**
Die Diagnose von Übergewicht und Adipositas erfolgt anhand des Body Mass Index (BMI). Von Übergewicht spricht man bei einem BMI ≥ 25 kg/m^2^, von Adipositas bei einem BMI ≥ 30 kg/m^2^ (Tab. 1)
Bei Personen mit ungewöhnlich großer (darunter SportlerInnen) oder kleiner Muskelmasse muss die Interpretation des BMI mit Vorsicht erfolgen
Eine Messung des Taillenumfangs sowie Bioimpedanzanalyse (BIA), wenn verfügbar, ist empfohlen zur Komplettierung der klinischen Untersuchung
**4.3. Abschätzung des Risikos für Adipositas-assoziierte Erkrankungen**
Die ausschließliche Verwendung des BMI ist für die Beurteilung der gesundheitlichen Relevanz von Übergewicht nicht ausreichend
Zur individuellen Risikoabschätzung sollen BMI, Taillenumfang und das Vorliegen von Begleiterkrankungen herangezogen werden (Abb. 1; Tab. 2)
Bei der Bestimmung des individuellen Gesamtgesundheitsrisikos sind zusätzlich weitere Risikofaktoren wie Ethnie, Alter, Geschlecht, Rauchen etc. zu berücksichtigen
**4.4. Spezifische Anamnese**
Zur Feststellung potenzieller Ursachen für Übergewicht/Adipositas und als Grundlage für die Entwicklung eines umfassenden strukturierten Behandlungsplans soll Folgendes erhoben werden: – Persönliche Einschätzung des eigenen Körpergewichts, der Adipositas-Diagnose und der Gründe für die Gewichtszunahme – Gewichtsverlauf – Bereits durchgeführte Gewichtsreduktionsversuche; welche Erfahrungen wurden damit gemacht? – Psychosozialer Stress, psychiatrische Störungen, Essstörungen – Familiäres und soziales Umfeld – Hinweise auf organische oder genetische Ursachen für Übergewicht/Adipositas – Familienanamnese hinsichtlich Übergewicht/Adipositas und damit assoziierten Komorbiditäten – Medikamentenanamnese (insbesondere im Hinblick als Ursache für Übergewicht/Adipositas); Möglichkeiten zur Optimierung der Medikation von Begleiterkrankungen (Tab. 2). – Inwieweit ist eine gewichtsreduzierende Intervention im Hinblick auf eine Verbesserung des individuellen Gesundheitszustands sinnvoll? – Sind Motivation und Bereitschaft abzunehmen gegeben?
**4.5. Indikationen zur therapeutischen Intervention**
*Lebensstilmaßnahmen* (s. Abschn. 4.7–4.9) sind indiziert bei – BMI ≥ 25 kg/m^2^ mit Begleiterkrankungen/Risikofaktoren (Tab. 2), die durch eine Gewichtsreduktion günstig beeinflusst werden können, oder – BMI ≥ 30 kg/m^2^ oder – BMI ≥ 25 kg/m^2^ bis < 30 kg/m^2^ ohne Begleiterkrankungen/Risikofaktoren (Tab. 2) können gewichtsreduzierende Maßnahmen empfohlen werden bei einem Leidensdruck der Betroffenen
*Pharmakologische Therapien* (s. Abschn. 4.10) sind indiziert bei – BMI ≥ 30 kg/m^2^ oder – BMI ≥ 27 kg/m^2^ mit Begleiterkrankungen/Risikofaktoren (Tab. 2)
*Bariatrisch-chirurgische Eingriffe *sind indiziert, wenn ein suffizienter Gewichtsverlust durch konservative Therapiemaßnahmen nicht erreicht werden kann – Bei Personen mit BMI ≥ 35 kg/m^2^, deren Gesundheit durch eine Gewichtsreduktion günstig beeinflusst werden kann – Bei Personen mit einem BMI ≥ 30 kg/m^2^ und Adipositas-assoziierten Komorbiditäten kann eine bariatrische Operation in Erwägung gezogen werden
**4.6. Lebensstilinterventionen**
Lebensstilmaßnahmen stellen die Basistherapie bei allen Personen mit Indikation zur gewichtsreduzierenden Intervention dar
Therapieziel ist ein Gewichtsverlust von 0,25–1 kg pro Woche bzw. von 5–10 % des Ausgangsgewichts innerhalb von 6 Monaten
Interventionen zur Lebensstiländerung sollen immer folgende Komponenten umfassen – Änderung der Essgewohnheiten, energiereduzierte Ernährung – Steigerung der körperlichen Aktivität – Unterstützende Maßnahmen zur Verhaltensänderung (s. Abschn. 4.11)
Durch die Maßnahmen soll ein Energiedefizit entstehen, d. h. der tägliche Energieverbrauch ist größer als die tägliche Energieaufnahme
Maßnahmen zur Lebensstiländerung sollen an die individuelle Situation (z. B. Fitnesslevel, Gesundheitszustand, aktueller Lebensstil) bzw. die Präferenzen der zu behandelnden Person angepasst werden. Das berufliche und persönliche Umfeld soll miteinbezogen werden
Die Lebensstiländerung soll dauerhaft sein
Die Betreuung muss jeweils durch Personen mit einer Ausbildung in den entsprechenden Gesundheitsberufen erfolgen
Die Betreuung kann individuell oder in Gruppen erfolgen
**4.7. Energiereduzierte Ernährung**
Vor Beginn soll Folgendes erhoben werden: – Essverhalten, Lebensmittelpräferenzen, Zusammensetzung und Kalorienmenge der derzeitigen Ernährung – Nahrungsmittelallergien und -unverträglichkeiten – Ausmaß der körperlichen Aktivität – Energiegrundumsatz, kalorimetrisch ermittelt oder geschätzt z. B. nach der Harris-Benedict-Formel (Tab. 3) – Gesamtumsatz, geschätzt auf Basis des Grundumsatzes, korrigiert um einen aktivitätsabhängigen Faktor (Tab. 3)
Durch die energiereduzierte Ernährung soll ein tägliches Energiedefizit von 500–600 kcal bezogen auf den Gesamtumsatz erreicht werden (Abb. 3)
Die Ernährung soll an die individuellen Ernährungsgewohnheiten und Nahrungsmittelpräferenzen und an das Risikoprofil und das Alter angepasst sein
Für die energiereduzierte Ernährung sind unterschiedliche Ernährungsformen gleich gut geeignet, sofern diese über einen ausreichenden Zeitraum zu einem Energiedefizit führen, ausgewogen sind und keine Gesundheitsschäden hervorrufen
Mögliche zusätzliche Interventionen zur Unterstützung der Einhaltung einer energiereduzierten Ernährung sind: – Ein strukturierter Ernährungsplan – Vorgepackte Portionen für die einzelnen Mahlzeiten – Mahlzeitenersatz durch Formula-Produkte – Alleinige Ernährung mit Formula-Produkten (täglicher Energiegehalt min. 800–1200 kcal). Diese sollen nicht routinemäßig, nur unter ärztliche Aufsicht und nur zeitlich begrenzt eingesetzt werden – Modifiziertes intermittierendes Fasten
Ernährungstherapie (inklusive Erstellung des Therapieplans und Monitoring) muss durch qualifizierte Ernährungsfachkräfte (z. B. DiätologInnen) in Form von Einzel- oder Gruppenberatung durchgeführt werden
Betroffene Personen sollen über Ziele, Prinzipien und praktische Aspekte einer Ernährungsumstellung informiert werden
**4.8. Steigerung von körperlicher Aktivität und Fitness**
Betroffene Personen sollen darüber informiert werden, dass eine Steigerung der körperlichen Aktivität auch unabhängig von einer Gewichtsreduktion günstige Effekte auf die Gesundheit hat
Für die Steigerung der körperlichen Aktivität sollen verständliche und realistische Ziele gesetzt werden
Auf die Unterschiede zwischen der Steigerung der Alltagsaktivität und Training soll hingewiesen werden
Die im Rahmen der Lebensstilintervention geplanten Aktivitäten sollen dem täglichen Leben angepasst sein (z. B. schnelles Gehen, Radfahren, Gartenarbeit, Stiegensteigen etc.)
Die körperliche Aktivität soll mit insgesamt mittlerer Intensität für zumindest 150–300 min pro Woche (idealerweise 5 bis 10 Einheiten zu je 30 min) erfolgen
Eine Kombination von Kraft- und Ausdauertraining wird empfohlen
Krafttraining soll unregelmäßige kurze intensive Einheiten und längere, kontinuierliche Übungen umfassen (mindestens 2 Einheiten pro Woche unter Einschluss aller großen Muskelgruppen)
Die Beratung (inklusive Erstellung des Therapieplans und Monitoring) soll nach Möglichkeit durch qualifizierte Fachkräfte in Bewegungs‑/Sporttherapie (Tab. 4, Empfehlungen 4.1) erfolgen
**4.9. Maßnahmen zur Verhaltensänderung**
Verhaltenstherapeutische Interventionen sollen von Personen aus Gesundheitsberufen mit psychotherapeutischer Kompetenz (z. B. klinische PsychologInnen, GesundheitspsychologInnen, PsychotherapeutInnen) im Rahmen von Einzel- oder Gruppensitzungen durchgeführt werden
Verhaltenstherapeutische Maßnahmen sollen an die individuelle Situation angepasst sein und verschiedene Elemente enthalten, wie z. B.: – Motivierende Gesprächsführung – Selbstmonitoring des Verhaltens und des Therapiefortschritts – Stimuluskontrolle – Setzen und Evaluieren von individuellen Zielen – Gesundes Essverhalten (u. a. Geschwindigkeit, situative Faktoren) – Strukturierte Essenspläne (in Zusammenarbeit mit DiätologInnen) – Soziale Unterstützung – Problembewältigungsstrategien – Anleitung zur Selbstbehauptung – Kognitive Restrukturierung – Festigung von Veränderungen – Rückfallprävention und -management
**4.10. Pharmakologische Gewichtsreduktion**
Eine pharmakologische Intervention zur Gewichtsreduktion soll nur mit Medikamenten erfolgen, die in dieser Indikation zugelassen sind (Tab. 5)
Missbräuchlicher Einsatz von Arzneimitteln mit gewichtsreduzierender Wirkung (z. B. Amphetamine, Diuretika, humanes Choriongonadotropin, Testosteron, Thyroxin, Wachstumshormone, Botox-Instillation) ist zu unterlassen
Vor dem Beginn einer pharmakologischen Intervention zur Gewichtsreduktion sollen die zu erwartenden Effekte, Risiken und Nebenwirkungen sowie die für die Behandlung notwendigen Kontrollen besprochen werden
**4.11. Evaluierung gewichtsreduzierender Interventionen**
Bei Personen, die das Therapieziel (Gewichtsreduktion um ≥ 5 % des Ausgangsgewichts) nach 3 bis 6 Monaten nicht erreicht haben, soll eine (Re‑)Evaluation des Behandlungsplans erfolgen. Dazu sollen BMI und Taillenumfang und nach Möglichkeit auch die Körperzusammensetzung (mittels BIA) erhoben und mit der letzten vorangegangenen Untersuchung verglichen werden
Medikamentöse Therapien haben zum Ziel, innerhalb von 3 bis 4 Monaten nach Erreichen der Zieldosis bzw. der höchsten verträglichen Dosierung eine Reduktion des Körpergewichts um zumindest 5 % zu erreichen [49–53]
**4.12. Gewichtserhaltende Interventionen**
Zur Prävention und zum Management von postinterventioneller Gewichtszunahme hat die Österreichische Adipositas Gesellschaft (2023) eigene Konsensusempfehlungen herausgegeben [17]

## Therapeutisches Management

Die komplexe Ätiologie von Übergewicht und Adipositas erfordert ein multifaktorielles therapeutisches Vorgehen, das diätetische Beratung, Anleitung zu körperlicher Aktivität, kognitive Verhaltenstherapie, psychologischen und sozialarbeiterischen Support und unterstützende medizinische Therapien (pharmakologisch, chirurgisch) umfasst. Für das praktische Management von Adipositas propagieren verschiedene Fachgesellschaften [31, 32] das „5A“-Modell, das ursprünglich für die Raucherentwöhnung entwickelt wurde, als evidenzbasierten Rahmen für verhaltenstherapeutische Interventionen [33]. Es umfasst die folgenden 5 Stufen (Abb. 3):

1. **„Ask“ – Ansprechen:** Zu Beginn muss geklärt werden, ob die/der PatientIn bereit ist, sich mit der eigenen Gewichtssituation auseinanderzusetzen. Aufgrund von Diskriminierung, Frustration, Schuldgefühlen oder negativen Erfahrungen im persönlichen oder medizinischen Umfeld reagieren Betroffene mitunter desinteressiert oder ablehnend, wenn sie auf die Notwendigkeit abzunehmen angesprochen werden. Voraussetzung für den Erfolg der Intervention ist aber, dass die betroffene Person zur Verhaltensänderung bereit ist. PatientInnen, die noch nicht mit einer gewichtsreduzierenden Therapie beginnen wollen oder können, sollten darauf hingewiesen werden, dass Beratung und Unterstützung für sie zur Verfügung stehen, und – ggf. durch Vereinbaren eines weiteren Beratungstermins – motiviert werden, die Gewichtsintervention zu einem späteren Zeitpunkt zu beginnen. Dazu sollten Kontaktdaten sowie Informationsmaterialien zu den Vorteilen von Gewichtsreduktion, gesunder Ernährung und erhöhter körperlicher Aktivität ausgehändigt werden. Der Umgang mit den Betroffenen und ihrem Umfeld soll respektvoll, empathisch und frei von Kritik und Vorurteilen erfolgen (Tab. 4, Empfehlungen 4.1).
2. **„Assess“ – Feststellen:** Diese Stufe umfasst eine umfassende allgemeine und spezifische Anamnese, die Diagnose der metabolischen Situation und der damit assoziierten Gesundheitsrisiken und die Dokumentation der Gewichthistorie inklusive Abnehmversuche. Parallel dazu sollen die subjektive Gesundheitswahrnehmung und die diesbezüglichen Zielvorstellungen der PatientInnen erhoben werden. Schließlich sollen die Ursachen des Übergewichts und der Faktoren, welche die bisherigen Bemühungen zur Lebensstiländerung behindert haben, identifiziert werden (Tab. 4, Empfehlungen 4.2–4.4).
3. **„Advise“ – Beraten:** Personen mit Übergewicht oder Adipositas sollen über ihr persönliches Gesundheitsrisiko aufgeklärt und dahingehend informiert werden, dass bereits moderater Gewichtsverlust den Gesundheitszustand spürbar verbessern kann, wobei auf die individuellen Wertvorstellungen der/des PatientIn fokussiert werden soll. Die PatientInnen sollen außerdem über die verschiedenen Behandlungsoptionen aufgeklärt werden. Die damit verbundenen Vorteile, Nachteile und Risiken und der von den PatientInnen zu leistende Beitrag zum Behandlungserfolg müssen verständlich gemacht werden.
4. **„Agree“ – Vereinbaren: **Im Sinn eines „Shared decision-making“-Prozesses soll der/die PatientIn beim Festsetzen der Therapieziele und bei der Planung der Maßnahmen zur Erreichung dieser Ziele eingebunden werden. Geplante Interventionen sollen besprochen und das Einverständnis der betroffenen Person eingeholt werden. Wichtig ist hier, dass die vereinbarten Ziele auch realistisch sind; die PatientInnen entsprechend zu beraten, ist auch Aufgabe der involvierten Fachkräfte.
5. **„Assist“ – Unterstützen:** Das Überwinden etablierter Verhaltensmuster und die Integration neuer Ernährungs- und Bewegungsgewohnheiten in den Alltag erfordern die Unterstützung aller involvierten Fachkräfte. Die PatientInnen sollen außerdem dahingehend beraten werden, welche Barrieren der Lebensstiländerung im Weg stehen und wie diese überwunden werden können. Partner bzw. Bezugspersonen sollen, soweit möglich, unterstützend in die Betreuung miteinbezogen werden (Tab. 4, Empfehlungen 4.1).

Im Folgenden wird auf das konservative Management (Lebensstilintervention, medikamentöse Therapie) von Übergewicht und Adipositas – ohne Berücksichtigung monogenetisch determinierter Ätiologien [11] – bei Erwachsenen eingegangen. Die Indikationen zur therapeutischen Intervention sind in Tab. 4, Empfehlungen 4.5, zusammengefasst. Zur Indikation und präoperativen Planung bariatrisch- bzw. metabolisch-chirurgischer Eingriffe sowie zum internistischen Management nach erfolgter Operation hat die Österreichische Adipositas Gesellschaft (2023) eigene Konsensusempfehlungen herausgegeben [34, 35]. Für die Durchführung metabolischer Operationen wird auf einschlägige Empfehlungen der Fachgesellschaften (z. B. [36]) verwiesen.

### Therapieziele

Übergeordnete Ziele der Behandlung von Übergewicht und Adipositas sind die Verbesserung des allgemeinen Gesundheitszustandes und der Lebensqualität sowie die Prävention bzw. positive Beeinflussung gewichtsassoziierter Erkrankungen und Risikofaktoren (Tab. 2). Die grundlegende therapeutische Strategie zur Erreichung dieser Ziele ist die Umkehr der energetischen Dysbalance und in der Folge eine anhaltend negative Energiebilanz. Zur Therapieverlaufskontrolle eignen sich die Gewichtsentwicklung (Abnahme in kg und %), der Taillenumfang (Abb. 1) als Surrogat für die viszerale Fettmenge und die Ermittlung der Körperzusammensetzung (z. B. mittels BIA) [37]. Der Behandlungserfolg bemisst sich vorrangig nicht an der Gewichtsabnahme, sondern an PatientInnen-zentrierten Outcomes.

**Figure 4:**
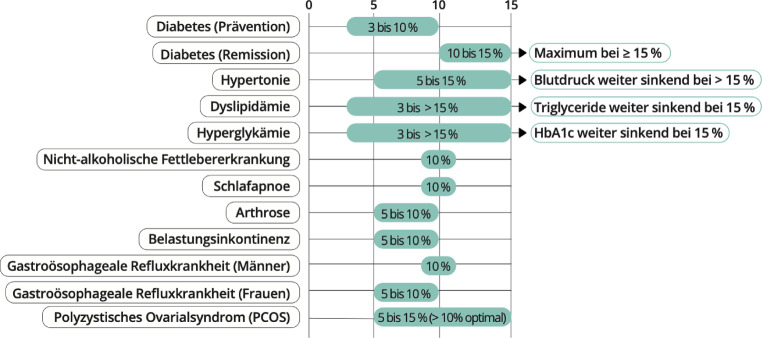

Die Therapieziele sind grundsätzlich individualisiert und unter Einbindung der betroffenen Person zu setzen, sie sollen realistisch und auf Nachhaltigkeit ausgelegt sein. Bei Personen ohne relevante Komorbiditäten kann, abhängig von den mentalen und psychosozialen Gegebenheiten, die Vermeidung einer weiteren Gewichtszunahme ein akzeptables Therapieziel darstellen. Klinisch relevante Gesundheitseffekte bis hin zur Verhinderung bzw. Remission gewichtsassoziierter Erkrankungen sind ab einer Gewichtsabnahme von 5–15 % des Ausgangsgewichts zu erwarten (Abb. 4; [38, 39]). Die Gewichtsintervention kann in diesem Fall auch dazu beitragen, die Intensität spezifischer Therapien (z. B. Antidiabetika, Antihypertensiva, Lipidsenker, Analgetika) zu reduzieren.

Neben der initialen Gewichtsreduktion ist die Verhinderung der erneuten Gewichtszunahme ein weiteres Therapieziel, das entsprechende Aufklärung und langfristige Betreuung erfordert [18].

### Ernährungstherapie

Die nachhaltige Veränderung der Ernährungsweise ist ein essenzieller Bestandteil jeder Gewichtsintervention und erfordert ein strukturiertes Vorgehen unter Einbindung spezialisierter Ernährungsfachkräfte, die zur Beratung von krankheitsverdächtigen und kranken Personen berechtigt sind (DiätologInnen, ErnährungswissenschaftlerInnen mit Ergänzungsstudium). Vor Therapiebeginn müssen die bisherigen Ernährungsgewohnheiten erfasst und allfällige Essstörungen (Tab. 2), die bei Menschen mit Adipositas signifikant häufiger auftreten als in der Allgemeinbevölkerung [40, 41], abgeklärt werden. Im nächsten Schritt wird mithilfe eines Ernährungsprotokolls das Ernährungsverhalten quantitativ (Welche Kalorienmengen werden zugeführt?) und qualitativ (Was, warum und in welchem Kontext wird gegessen?) erfasst. Dadurch können Problembereiche (z. B. der Energiegehalt vermeintlich gesunder Lebensmittel wie Fruchtsäfte) identifiziert und bewusst gemacht werden. Zudem liefert das Ernährungsprotokoll Ansatzpunkte für die kognitive Verhaltenstherapie, um Ernährungsmuster wie kompensatorisches oder emotionales Essverhalten aufzubrechen [26, 42].

Voraussetzung für die Gewichtsreduktion ist eine negative Energiebilanz bezogen auf den aktuellen Energiebedarf (Gesamtumsatz; Tab. 3 und 4, Empfehlungen 4.7). Kalorienrestriktionen müssen individualisiert unter Einbezug von Ernährungsgewohnheiten, dem aktuellen körperlichen Aktivitätslevel, Komorbiditäten und vorangegangenen Diätversuchen festgelegt werden. Eine Reduktion der aktuellen Energiezufuhr um 15–30 % ist anzustreben [26]. Dabei kann bei einem Energiedefizit von 500–600 kcal pro Tag mit einer durchschnittlichen Gewichtsreduktion von 0,5 kg/Woche (2 kg/Monat) gerechnet werden [43].

Allgemein akzeptierte Maßnahmen zur Reduktion der Energiezufuhr inkludieren die Beschränkung der Portionen und Portionsgrößen pro Mahlzeit, die Verwendung weniger energiedichter Nahrungsmittel mit höherem Ballaststoffanteil bei verringertem Gehalt an Fett (v. a. gesättigten Fettsäuren) und Kohlenhydraten (v. a. raffinierte Monosaccharide und zuckerhaltige Getränke). Diese Vorgaben können z. B. im Rahmen von balancierten Ernährungsformen wie der „mediterranen Ernährungsweise“ umgesetzt werden. Die Studienlage zur gewichtsreduzierenden Effizienz spezifischer Ernährungsformen ist uneindeutig. Für den Gewichtseffekt entscheidend ist aber nicht so sehr die Makronährstoffzusammensetzung („low carb“, „low fat“, „high protein“ etc.) als die nachhaltige Energiereduktion und damit auch die Akzeptanz und Umsetzbarkeit der Ernährungsform. Somit sind persönliche Präferenzen, das soziale Umfeld, finanzielle und zeitliche Ressourcen und außerdem Komorbiditäten und Risikofaktoren bei der Anleitung zur Ernährungsumstellung miteinzubeziehen. Der Einsatz von „Formula-Diäten“ mit niedrigem („low calory diet“ [LCD]; 800–1200 kcal/Tag) oder sehr niedrigem („very low calory diet“ [VLCD]; < 800 kcal/Tag) Kaloriengehalt kann in bestimmten klinischen Situationen angebracht sein. Die VLCD ist jedoch nicht für schwangere oder stillende Frauen, ältere Menschen sowie für Kinder und Jugendliche geeignet [26, 42, 43].

Die Ernährungstherapie sollte entsprechend multidisziplinär durch qualifizierte Fachkräfte durchgeführt werden.

### Bewegungstherapie

Körperliche Aktivität unterstützt die Ernährungstherapie zur Negativierung der Energiebilanz einerseits durch die Energie, die für die Muskelarbeit aufgewendet wird, andererseits durch Steigerung des Grundumsatzes (Tab. 3). Zu unterscheiden ist die Steigerung körperlicher Aktivität im Alltag von gezielten Trainingsmaßnahmen zur Verbesserung der Muskelmasse und der kardiorespiratorischen Fitness [23, 42]. Vermehrte Alltagsaktivität wirkt sich positiv auf das Wohlbefinden und das Selbstwertgefühl aus, erleichtert das Überwinden etablierter Verhaltensmuster und den Einstieg in die Trainingstherapie. Die Steigerung der kardiorespiratorischen Fitness als Folge von Ausdauertraining verbessert unabhängig vom Effekt auf das Körpergewicht den Glukose- und Lipidstoffwechsel und reduziert das kardiovaskuläre Risiko [43]. Krafttraining erhöht durch Zunahme der Muskelmasse den Grundumsatz, wirkt stärker als Ausdauertraining auf das Körpergewicht und verbessert die Gelenk- und Wirbelsäulenstabilität [44].

Im Rahmen der Lebensstilintervention sollte der Umfang der körperlichen Aktivität zumindest 150–300 min pro Woche bei mittlerer Aktivität, aufgeteilt auf 5 bis 10 Einheiten zu je 30 min, betragen (Tab. 4, Empfehlungen 4.8). Für eine effiziente Gewichtsreduktion müssen pro Woche 1000–2000 kcal an Bewegungskalorien verbraucht werden. Ausdauer- und Krafttraining sollen nach Möglichkeit kombiniert werden. Krafttraining ist zu bevorzugen, wenn aus Gewicht- und Konditionsgründen ein sinnvolles Ausdauertraining nicht möglich ist. Insbesondere bei älteren und kardiorespiratorisch eingeschränkten Personen ist Krafttraining aufgrund des geringeren Trainingsumfanges und -aufwandes zudem einfacher umzusetzen [45].

Die konkrete Ausgestaltung der Bewegungstherapie (Auswahl der geeigneten Bewegungsform; Frequenz, Intensität und Dauer der Aktivität) muss an die individuellen Anforderungen, Möglichkeiten und Fähigkeiten der PatientInnen angepasst und durch Fachkräfte, die zur medizinischen Trainingstherapie o. Ä. befugt sind (z. B. PhysiotherapeutInnen, SportwissenschaftlerInnen mit entsprechender Weiterbildung), geplant und überwacht werden. Besonderes Augenmerk ist auf die Vermeidung von Verletzungen zu legen, da verletzungsbedingte Pausen bereits erzielte Trainingsfortschritte wieder zunichtemachen können. Zu achten ist darüber hinaus auf die Umsetzbarkeit der geplanten Aktivitäten im Alltag. Bewegungsanleitungen sollen möglichst detailliert besprochen werden. Im Beratungsgespräch soll vermittelt werden, welche konkreten Ziele mit den einzelnen Maßnahmen verfolgt werden, und welche Effekte dadurch von den PatientInnen erwartet werden können [42, 45].

Die Bewegungstherapie sollte entsprechend multidisziplinär durch qualifizierte Fachkräfte durchgeführt werden.

### Verhaltenstherapie

Verhaltenstherapeutische Unterstützung ist für die nachhaltige Gewichtskontrolle wesentlich. Die Planung und Umsetzung der Interventionen soll von Personen aus Gesundheitsberufen mit psychotherapeutischer Kompetenz (z. B. klinische PsychologInnen, GesundheitspsychologInnen, PsychotherapeutInnen) durchgeführt werden (Tab. 4, Empfehlungen 4.9; [46]). In der psychologischen Aufklärung (Psychoedukation) soll den PatientInnen das nötige Wissen vermittelt werden, um sich auf realistische Therapieziele einigen und diese langfristig verfolgen zu können. Durch Selbstbeobachtung und Verhaltensanalyse werden problematische Verhaltensweisen genauer erfasst. Dies kann durch Strategien zur Reizkontrolle (Stimuluskontrollstrategien), Stressbewältigung/Stressmanagement und/oder sozialer Unterstützung erfolgen. Elemente der kognitiven Therapie helfen, problematischer Schemata zu rekonstruieren und Probleme zu lösen [47, 48].

Verhaltenstherapeutische Maßnahmen sollen an die individuelle Situation angepasst sein und eine multifaktorielle Strategie verfolgen (Tab. 4, Empfehlungen 4.9; [48]). Die Planung und Umsetzung der Interventionen soll multidisziplinär von Personen aus Gesundheitsberufen mit psychotherapeutischer Kompetenz (z. B. klinische PsychologInnen, GesundheitspsychologInnen, PsychotherapeutInnen) durchgeführt werden.

### Medikamentöse Therapie

Von der Europäischen Arzneimittelagentur (EMA) sind derzeit 4 Wirkstoffe bzw. Wirkstoffkombinationen für die Gewichtskontrolle zugelassen (Tab. 5): die GLP-1-RA Liraglutid 3 mg und Semaglutid 2,4 mg und außerdem Orlistat 60 und 120 mg sowie Bupropion/Naltrexon 78/7,2 mg. Die Indikation umfasst jeweils die unterstützende Gewichtskontrolle zusätzlich zu Lebensstilmaßnahmen bei Personen mit einem BMI ≥ 30 kg/m^2^ oder aber mit einem BMI ≥ 27 kg/m^2^ und zumindest einer gewichtsassoziierten Erkrankung (Orlistat: BMI ≥ 28 kg/m^2^ mit begleitenden Risikofaktoren). Liraglutid und Semaglutid sind ab 12 Jahren zugelassen, während der Einsatz von Orlistat und Bupropion/Naltrexon auf Erwachsene beschränkt ist. Mit Ausnahme von Orlistat 60 mg sind alle Therapien rezeptpflichtig. Orlistat 120 mg soll in der Schwangerschaft mit Vorsicht angewendet werden, ansonsten sind alle genannten Therapien bei schwangeren und bei stillenden Frauen nicht empfohlen bzw. kontraindiziert.

**Table 5:** 

Orlistat 120 mg (Xenical®, generisch), Orlistat 60 mg (generisch; rezeptfrei)
Bupropion/Naltrexon 78/7,2 mg (Mysimba®)
Liraglutid 3 mg (Saxenda®)
Semaglutid 2,4 mg (Wegovy®)

Alle von der EMA zugelassenen gewichtsreduzierenden Therapien müssen hinsichtlich des Therapieansprechens evaluiert werden: Orlistat 120 mg, Liraglutid und Semaglutid müssen nach 12 Wochen unter maximaler Dosierung, Naltrexon/Bupropion nach 16 Wochen abgesetzt werden, sofern der Gewichtsverlust nicht ≥ 5 % des Ausgangsgewichts beträgt. In der Selbstmedikation mit Orlistat 60 mg soll ärztlicher Rat eingeholt werden, wenn es innerhalb von 12 Wochen zu keiner Gewichtsreduktion kommt [49–53].

#### Orlistat

ist ein spezifischer Inhibitor der gastrointestinalen Lipasen und hemmt die Hydrolyse von Nahrungsfetten in resorbierbare freie Fettsäuren im Magen und im oberen Dünndarm. Die Behandlung erfolgt in Kombination mit einer leicht hypokalorischen, fettreduzierten Ernährung, bis zu 3‑mal täglich zu den Hauptmahlzeiten [48, 50]. Klinische Studien weisen für Orlistat eine mittlere Gewichtsreduktion um 3,2 % des Ausgangsgewichts aus; die Wahrscheinlichkeit (ausgedrückt als Odds-Ratio), ≥ 5 % an Gewicht zu verlieren, liegt nach Metaanalyse der Studiendaten bei 2,7 [54]. Häufige Nebenwirkungen betreffen v. a. den Gastrointestinaltrakt: Bauchschmerzen und Diarrhö mit fettigem/öligem Stuhl [49, 50].

#### Bupropion/Naltrexon

Bupropion ist ein ursprünglich zur antidepressiven Behandlung und zur Raucherentwöhnung eingesetzter Inhibitor der neuronalen Dopamin- und Noradrenalin-Wiederaufnahme, Naltrexon ein in der Therapie von Alkohol- und Opiatabhängigkeiten etablierter Opioidrezeptorantagonist. Die Wirkung der Fixkombination beruht auf der Aktivierung von anorexigenen Neuronen im Hypothalamus [51]. In klinischen Studien reduzierte Bupropion/Naltrexon das Gewicht im Durchschnitt um 4,1 % des Ausgangsgewichts, die Odds-Ratio für eine Reduktion um ≥ 5 % betrug 5 [54]. Zu den häufigsten Nebenwirkungen zählen Übelkeit, Erbrechen, Obstipation sowie Schwindel und Mundtrockenheit. Bei nicht kontrollierter Hypertonie und bei schwerwiegenden psychiatrischen Erkrankungen darf das Präparat nicht verwendet werden [51].

#### Liraglutid

Liraglutid ist ein rekombinantes Analogon des humanen Inkretinhormons GLP‑1 und in der Dosierung bis zu 3 mg/Tag zur Behandlung von Adipositas zugelassen [52]. In supraphysiologischer Konzentration verlangsamen GLP‑1 und GLP-1-Rezeptoragonisten die Magenmotilität und dämpfen über die Modulation neuronaler Regelkreise im Hypothalamus den Appetit und das Hungergefühl [55]. In den Zulassungsstudien im Rahmen des SCALE-Studienprogrammes kam es unter der Dosis von 3 mg/Tag innerhalb eines Jahres zu einem durchschnittlichen Gewichtsverlust von 5,7–8,0 % des Ausgangsgewichts. Außerdem zeigte eine Studie bei Menschen mit Prädiabetes, dass durch Liraglutid 3 mg das relative Risiko zur Entwicklung eines Typ-2-Diabetes um 79 % reduziert werden kann [56]. Liraglutid wird 1‑mal täglich subkutan injiziert. Zu den häufigsten Nebenwirkungen v. a. zu Therapiebeginn in den ersten 12 Wochen zählen Übelkeit, Erbrechen, Diarrhö, aber auch Obstipation, zudem können Cholelithiasis und Cholezystitis gehäuft auftreten. Bei Verdacht auf Pankreatitis ist die Behandlung abzusetzen und bei bestätigter Pankreatitis nicht wieder aufzunehmen [52].

#### Semaglutid

Semaglutid ist wie Liraglutid ein GLP-1-Analogon mit hoher Sequenzhomologie zum humanen Inkretinhormon, weist aufgrund weiterer Modifikationen aber eine deutlich längere Halbwertszeit und stabilere Plasmaspiegel auf und wird 1‑mal wöchentlich bis zu einer Maximaldosis von 2,4 mg subkutan injiziert [53]. Die Metaanalyse der Zulassungsstudien ergab eine mittlere Gewichtsreduktion von 11,4 % des Ausgangsgewicht und eine Odds-Ratio von 9,8 für eine Gewichtsabnahme um ≥ 5 % [54]. In den Zulassungsstudien erreichten zudem 51–64 % der PatientInnen mit Übergewicht oder Adipositas (26 % der PatientInnen mit Typ-2-Diabetes und BMI ≥ 27 kg/m^2^) eine Reduktion um ≥ 15 % des Ausgangsgewichts [57]. Semaglutid reduzierte in niedrigerer Dosierung (1 mg) bei Personen mit Typ-2-Diabetes schwerwiegende kardiovaskuläre Ereignisse [58]. Die Ergebnisse einer kardiovaskulären Outcome-Studie mit Semaglutid 2,4 mg bei Menschen mit Adipositas (SELECT; NCT03574597) werden Ende 2023 erwartet. Das Nebenwirkungsspektrum von Semaglutid und ebenso das Vorgehen bei Verdacht auf bzw. bei bestätigter Pankreatitis entspricht jenem bei Liraglutid [52, 53].

#### Ausblick

Eine Erweiterung der therapeutischen Optionen ist aus heutiger Sicht v. a. aus der Gruppe der dualen Inkretinagonisten zu erwarten. Der für die Diabetestherapie bereits zugelassene duale GLP-1/GIP-Agonist Tirzepatid erreichte in Adipositasstudien Gewichtsabnahmen um bis zu 21 % des Ausgangsgewichts [59]. Weitere duale Rezeptoragonisten (z. B. GLP-1/Glukagon; GLP-1/Amylin, wie z. B. Semaglutid/Cagrilintid) [60] bzw. auch Triple-Agonisten (GLP-1/GIP/Glukagon, wie z. B. Retatrutid) [61] sind in Entwicklung.

### Therapie von Begleiterkrankungen

Das Management von Begleiterkrankungen (Tab. 2) erfolgt unabhängig von der gewichtsreduzierenden Therapie entsprechend den jeweiligen Richtlinien. Medikamente mit gewichtsreduzierender bzw. gewichtsneutraler Wirkung sollen, soweit möglich und sinnvoll, gegenüber Medikamenten mit ungünstigem Einfluss auf das Körpergewicht bevorzugt werden. Die PatientInnen sollen hinsichtlich der Gewichtseffekte der verfügbaren Therapieoptionen beraten werden.

## Reevaluierung der Gewichtsintervention

Gewichtsreduzierende Interventionen sollen grundsätzlich nach 3 bis 6 Monaten evaluiert und, sofern die angestrebten Therapieziele nicht erreicht wurden, angepasst werden (Tab. 4, Empfehlungen 4.11). Neben dem Verlauf von BMI und Taillenumfang sind Änderungen der klinischen Situation (Risikofaktoren, Begleiterkrankungen) in die Evaluation miteinzubeziehen. Zu bewerten ist außerdem, welche Kalorienmengen durchschnittlich aufgenommen und verbraucht wurden (Ist – Soll), inwieweit die vereinbarten Lebensstiländerungen umgesetzt wurden und welche Gründe aus Sicht der Betroffenen für das Nichterreichen der Therapieziele verantwortlich sind bzw. welche Barrieren der Einhaltung des Behandlungsplans entgegenstehen.

Basierend auf den Ergebnissen der Reevaluierung, wird der Behandlungsplan optimiert. Hinsichtlich der Festigung der Lebensstiländerung bzw. der Einhaltung des Behandlungsplans soll Unterstützung angeboten werden. Weitere Hinweise zur langfristigen Behandlungsplanung finden sich in den ÖAG-Empfehlungen „Prävention und Management von postinterventioneller Gewichtszunahme“ (2023) [18].

## Interessenkonflikt

Die Autor-/innen weisen auf folgende Beziehungen hin. J.M. Brix hat Honorare für Advisory Boards und Vorträge sowie Kongressunterstützungen von AstraZeneca, Boehringer Ingelheim, Eli Lilly und Novo Nordisk erhalten. K. Aydinkoc-Tuzcu hat in den letzten drei Jahren Forschungsunterstützungen, Honorare für Beratungstätigkeit, Honorare für Vortragstätigkeit von Eli Lilly, Novo Nordisk, Böhringer Ingelheim, Abbott erhalten. Zusätzlich gibt Kadriye Aydinkoc-Tuzcu an, dass für sie persönlich kein Interessenkonflikt vorliegt. P. Beckerhinn hat in den letzten drei Jahren Honorare für Vortragstätigkeit von der Firma Johnson & Johnson erhalten. A. Cavini hat in den letzten drei Jahren keine Forschungsunterstützungen, keine Honorare für Beratungstätigkeit, aber vereinzelt Honorare für Vortragstätigkeit von Nutricia erhalten. M. Clodi hat in den letzten drei Jahren Forschungsunterstützungen, Honorare für Beratungstätigkeit, Honorare für Vortragstätigkeit von Eli Lilly, Astra Zeneca, Boehringer Ingelheim, Sanofi, Novo Nordisk, Amgen erhalten. D.M. Felsenreich hat Honorare für Vortragstätigkeit von Österreichischer Adipositas Gesellschaft und Österreichisches Akademisches Institut für Ernährungsmedizin erhalten. B. Göbel hat in den letzten drei Jahren *keine* Forschungsunterstützungen, Honorare für Beratungstätigkeit oder Honorare für Vortragstätigkeit erhalten, nur Kongressteilnahmen wurden von der Firma Eli Lilly übernommen. F. Hoppichler hat in den letzten drei Jahren Forschungsunterstützungen, Honorare für Beratungstätigkeit, Honorare für Vortragstätigkeit von Eli Lilly, Böhringer Ingelheim, Novo Nordisk, Sanofi und Astra Zeneca erhalten. J. Huber hat in den letzten drei Jahren Honorare für Beratungstätigkeit, Honorare für Vortragstätigkeit und Unterstützung für die Teilnahme bei Fortbildungen von folgenden Firmen erhalten: Boehringer-Ingelheim, Astra Zeneca, Novo Nordisk, Eli Lilly, Sanofi, Viatris, MSD. B.K. Itariu hat in den letzten drei Jahren Honorare für Beratertätigkeit und Vortragstätigkeit von AMGEN, Böhringer-Ingelheim und Novo Nordisk erhalten. B. Jandrasitz hat in den letzten drei Jahren keine Forschungsunterstützungen, Honorare für Beratungstätigkeit, Honorare für Vortragstätigkeit erhalten. F.W. Kiefer hat Honorare für Beratungstätigkeit, Vortragstätigkeiten und Honorare für Astra Zeneca, Boehringer, Lilly, Novartis, Novo Nordisk und Sanofi erhalten. G. Köhler hat in den letzten drei Jahren Forschungsunterstützungen, Honorare für Beratungstätigkeit, Honorare für Vortragstätigkeit von Astra Zeneca, Austro Control, Amgen, Böhringer Ingelheim, Eli Lilly, Medizinische Universität Graz, Novo Nordisk, Roche Diagnostics, Sanofi Aventis erhalten. B. Ludvik erhielt Honorare für Vorträge und Advisory Boards sowie Forschungsuntertützung von Eli Lilly, Novo Nordisc und Orexigen. G. Prager hat in den letzten drei Jahren einen Educational Grant von Medtronic und Johnson&Johnson erhalten, und Speaker Fees von Medtronic. C. Ress hat in den letzten drei Jahren Forschungsunterstützungen, Honorare für Beratungstätigkeit, Honorare für Vortragstätigkeit von AstraZeneca, Novo Nordisk, Pfizer, Boehringer Ingelheim, Amryt und Sanofi erhalten. C. Schelkshorn hat Honorare für Vorträge und Kongressunterstützung von folgenden Firmen erhalten: Böhringer Ingelheim, Lilly Pharma, Novo Nordisk, Sanofi Pharma, Novartis, Astra Zeneca, Abbott Diabetes, Advisory Board: Novo Nordisk, Böhringer Ingelheim/Eli Lilly/Astra Zeneca. T. Scherer hat in den letzten drei Jahren Forschungsunterstützungen, Honorare für Beratungstätigkeit, Honorare für Vortragstätigkeit von folgenden Firmen erhalten: Vortragshonorare: Amicus, Alnylam, AstraZeneca, BMS, Merck, Novonordisk, Böhringer, Sanofi, Shire, Beratertätigkeit Amicus, Sanofi, Amryt, Forschungsgrants: Amryt, Sanofi. H. Sourji hat in den letzten drei Jahren Forschungsunterstützungen (an die MedUni Graz), Honorare für Beratungstätigkeit, Honorare für Vortragstätigkeit von Amarin, Amgen, AstraZeneca, Bayer, Böhringer Ingelheim, Daiichi Sankyo, Eli Lilly, MSD, Novartis, NovoNordisk, Sanofi erhalten. L. Stechemesser hat in den letzten drei Jahren Forschungsunterstützungen, Honorare für Beratungstätigkeit, Honorare für Vortragstätigkeit von Abbott, Amgen, Boehringer Ingelheim, Daiichi Sankyo, Dexcom, Eli Lilly, Medtronic, Medtrust, Mylan, Novartis, NovoNordisk und Sanofi erhalten. T. Stulnig hat in den letzten drei Jahren Forschungsunterstützungen, Honorare für Beratungstätigkeit, Honorare für Vortragstätigkeit von Amgen, AstraZeneca, Boehringer-Ingelheim, Daiichi-Sankyo, Eli Lilly, Novartis, Novo Nordisk, Sobi und Sanofi erhalten. H. Toplak hat in den letzten drei Jahren Honorare für Klinische Forschung, Beratertätigkeit und Vortragshonorare von Novo Nordisk erhalten. A. Vonbank hat in den letzten drei Jahren Forschungsunterstützungen, Honorare für Beratungstätigkeit, Honorare für Vortragstätigkeit von Amgen, Eli Lilly, Amarin, Novo Nordisk, Bayer, Daichii Sankyo, Sandoz, Novartis und Böhringer Ingelheim erhalten. D. Weghuber hat in den letzten drei Jahren Forschungsunterstützungen, Honorare für Beratungstätigkeit, Honorare für Vortragstätigkeit von Novo Nordisk A/S erhalten.

B. Andersen, A. Brossard-Eitzinger, C. Ciardi, M. Eichner, B. Erlacher, M. Fahrnberger, C. Francesconi, E. Hölbing, S.L. Huber, R. Kruschitz, A. Malzner, A. Moosbrugger, A. Öfferlbauer-Ernst, V. Parzer, M. Resl und M. Wakolbinger geben an, dass kein Interessenkonflikt besteht.
